# Current therapies and future prospective for locally aggressive mesenchymal tumors

**DOI:** 10.3389/fonc.2023.1160239

**Published:** 2023-07-21

**Authors:** Alessandra Maleddu, Jessica Zhu, Michael Roy Clay, Breelyn Ann Wilky

**Affiliations:** ^1^ Department of Medicine, University of Colorado School of Medicine, Aurora, CO, United States; ^2^ Department of Pathology, University of Colorado School of Medicine, Aurora, CO, United States

**Keywords:** desmoid fibromatosis, giant cell tumor of bone, tenosynovial giant cell tumor, locally aggressive mesenchymal tumors, malignant giant cell tumor of bone, metastatic giant cell tumor of bone, tyrosine kinase inhibitors, γ-secretase inhibitors

## Abstract

Locally aggressive mesenchymal tumors comprise a heterogeneous group of soft tissue and bone tumors with intermediate histology, incompletely understood biology, and highly variable natural history. Despite having a limited to absent ability to metastasize and excellent survival prognosis, locally aggressive mesenchymal tumors can be symptomatic, require prolonged and repeat treatments including surgery and chemotherapy, and can severely impact patients’ quality of life. The management of locally aggressive tumors has evolved over the years with a focus on minimizing morbid treatments. Extensive oncologic surgeries and radiation are pillars of care for high grade sarcomas, however, play a more limited role in management of locally aggressive mesenchymal tumors, due to propensity for local recurrence despite resection, and the risk of transformation to a higher-grade entity following radiation. Patients should ideally be evaluated in specialized sarcoma centers that can coordinate complex multimodal decision-making, taking into consideration the individual patient’s clinical presentation and history, as well as any available prognostic factors into customizing therapy. In this review, we aim to discuss the biology, clinical management, and future treatment frontiers for three representative locally aggressive mesenchymal tumors: desmoid-type fibromatosis (DF), tenosynovial giant cell tumor (TSGCT) and giant cell tumor of bone (GCTB). These entities challenge clinicians with their unpredictable behavior and responses to treatment, and still lack a well-defined standard of care despite recent progress with newly approved or promising experimental drugs.

## Introduction

Desmoid fibromatosis, giant cell tumor of bone and tenosynovial giant cell tumor are three distinct locally aggressive mesenchymal tumors with unpredictable behavior and absent to low tendency for malignancy (1). Historically, DF, GCTB and TSGCT have been managed following paradigms of treatment for high grade sarcomas with aggressive surgeries and radiation treatment. However, important differences with respect to epidemiology, biology and prognosis between locally aggressive tumors and sarcomas have led to substantial changes in management over the last few years. Specifically, DF, GCTB and TSGCT affect predominantly young adults and, despite being locally aggressive and often highly symptomatic, have excellent prognosis (2–4). For all these reasons, and for the high rate of local recurrence, aggressive surgeries are no longer recommended. Similarly, radiation therapy is very rarely used nowadays for the risk of both malignant transformation and secondary cancer. The dismissal of aggressive treatments, the introduction of new drugs, the advancements in local treatment techniques, and better understanding of tumor biology have revolutionized the management of DF, GCTB TSGCT (5, 6). These diseases are now regarded more as chronic conditions in need of long-term symptoms and disease control without quality-of-life detriment. Patient associations and the expanding use of patient-reported outcome measures (PROMs) have largely contributed advancement in understanding the many physical, psychosocial, and practical challenges that patient encounter (7, 8).

## Desmoid-type fibromatosis

Desmoid-type fibromatosis (DF), also known as aggressive fibromatosis, is a monoclonal fibroblastic neoplasm characterized by an infiltrative and locally aggressive growth pattern, high rates of post-surgical recurrence, and no metastatic potential (1).

***Epidemiology.*** The incidence of DF is low with around 5 new cases per million people per year, with a peak between the 3^rd^ or 4^th^ decade of life and higher incidence in female patients (2).

**Figure 1:**
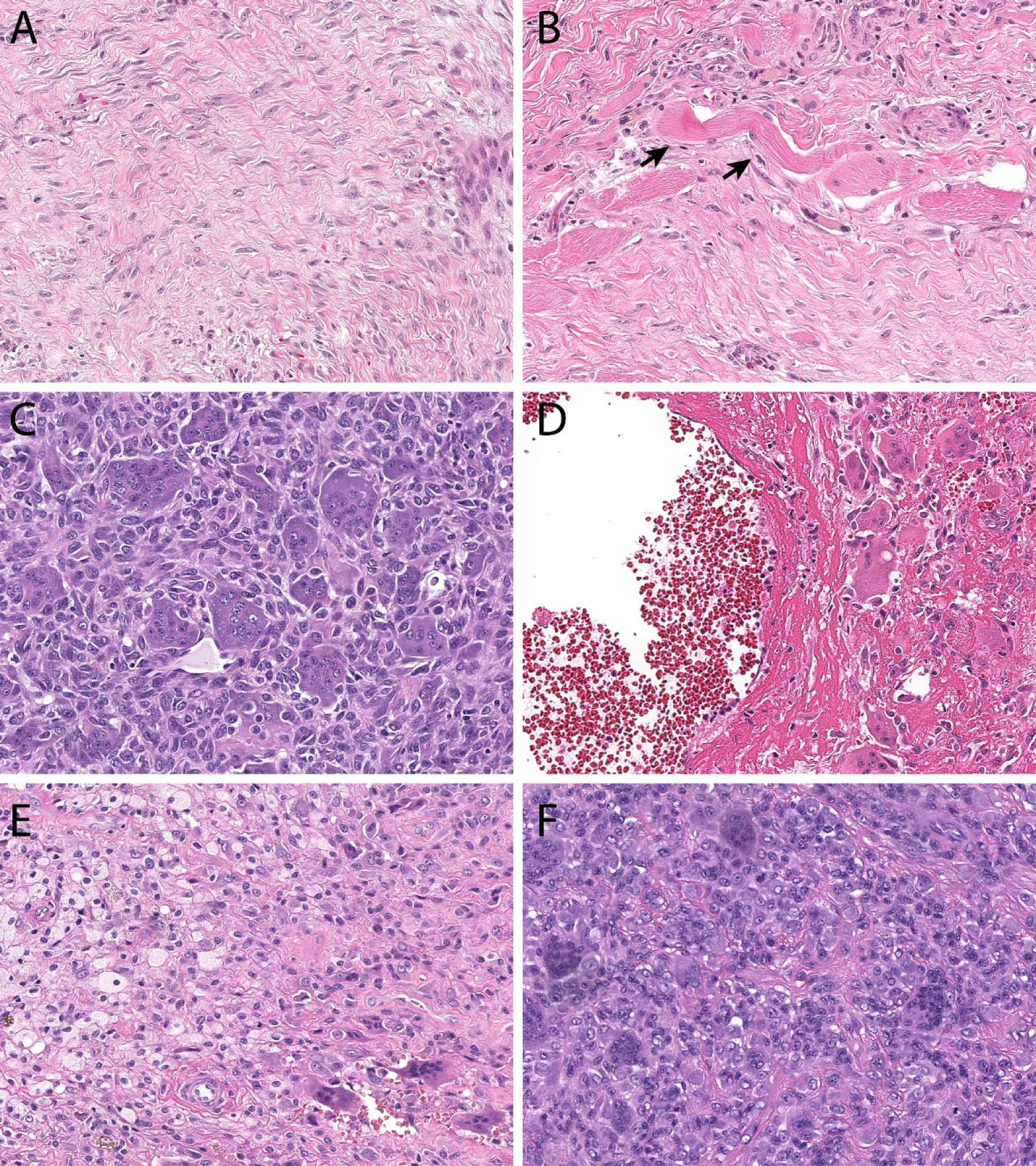
Histopathologic features. Desmoid-type fibromatosis **(A)** contains bland spindled cells arranged in a vague fascicular pattern. They often demonstrate skeletal muscle invasion (**B**, skeletal muscle fibers at black arrows), a finding that correlates with locoregional recurrence and incomplete excision. Giant cell tumor of bone **(C)** is comprised of monotonous mononuclear cells and an even distribution of osteoclastic giant cells. Both cell populations display similar nuclear features. In many instances, secondary aneurysmal bone cyst change **(D)** can be seen and can mask the underlying features. Tenosynovial giant cell tumor **(E)** is comprised of an admixture of foamy macrophages, osteoclastic giant cells, and inflammation. Monomorphic variants **(F)** can display increased cellularity, mimicking a sarcoma.

***Histopathology.*** Histologically, DF rarely cause diagnostic confusion, and are reliably comprised of bland hypochromatic spindled cells arranged in a densely fibrotic stroma (**Figures 1A, B**).

***Etiopathogenesis.*** The etiopathogenesis of DF is not completely understood and likely multifactorial. Approximately 85-90% of DF cases are sporadic and harbor a mutation of the gene encoding the beta catenin protein, CTNBB1; whilst the remaining 5-10% of DF harbor an APC gene mutation and arise in the context of Familial Adenomatous Polyposis Syndrome (FAP) or attenuated FAP syndrome (9, 10). Key events in DF tumorigenesis are the genetic alterations of CTNNB1 or APC in sporadic or hereditary cases, respectively. Both mutations lead to constitutive activation of the Wnt/β-catenin pathway. In addition, Notch target genes have been shown to be overexpressed in DT and to engage in cross-talk with the Wnt/β-catenin signaling pathway, providing alternative potential therapeutic targets (11). Trigger events for tumorigenesis are thought to be a recent trauma, surgery, or pregnancy (12, 13).

***Genetic testing.*** Molecular testing is encouraged as part of the diagnostic workup as virtually all DF harbor mutually exclusive mutations of either the CTNNB1 or APC genes (9, 14).

***Clinical presentation.*** Clinically, DF can occur in any anatomic location. The vast majority of sporadic DF arise in the limbs, chest, and abdominal wall, while the intra-abdominal and head and neck location are less frequent. A previous surgery, trauma or recent pregnancy are common anamnestic findings and are frequently associated with *de novo* DF growth or progression of disease (1, 12, 13). FAP-associated DF harbor APC mutations can be multifocal and are frequently intra-abdominal. The diagnosis of APC mutated DF warrants FAP workup with colonoscopy and germline testing (9).

***Natural history.*** The natural history of DF is unpredictable and can vary widely between patients; presenting symptoms depend on the growth rate and anatomic location of the tumor. Tumors can elicit severe symptoms when abutting nerves or vessels, or cause severe damage encompassing or invading intra-abdominal organs such as the bowel (15). In the last several years, the treatment approach has evolved considerably with emerging prospective evidence that long term stable disease and even spontaneous regression can occur in up to 20% of DF, even after an initial phase of growth (15–19).

## Treatment

There is no standard of care for DF, which have been historically managed using similar paradigms to high grade sarcomas, with attempts at complete resection even at the cost of morbid surgeries, and various cytotoxic chemotherapies for unresectable tumors (15–19). The Desmoid Tumor Working Group (DTWG) is an international team of desmoid fibromatosis experts that in 2020 has issued evidence-based consensus guidelines with the aim of improving quality of care and patient’s outcome worldwide (9).

***Active surveillance.*** A “watch and wait” approach defined as “active surveillance” has been recommended by the DTWG for newly diagnoses patients, when the clinical presentation allows it, in view of the unpredictable behavior of DF and the high rate of spontaneous regression (9). Treatment initiation should be based on clear radiographic progression or emerging clinical symptoms (9). Patients managed with active surveillance should be monitored with imaging at 1 or 2 months from diagnosis then every 3 to 6 months. Progression in a single assessment in the absence of symptoms and when the tumor is in a non-critical location is not indication for treatment. Ideally, patients on active surveillance should be evaluated by an expert physician at a reference center for DF as the risk of progression may be high for large tumors (9).

When disease progression has been documented in at least two subsequent imaging assays, in the presence of worsening symptoms and for tumor arising in anatomical-critical locations, treatment should be considered. Systemic therapies should be favored over upfront surgical resection, which is now discouraged and reserved to few, selected cases due to preponderance of incomplete initial resections and frequent recurrences (9, 16–18, 20, 21).

## Locoregional treatments

While surgery and radiation therapy (RT) are less and less employed, locoregional treatments such as cryoablation and high intensity focused ultrasound ablation have gained considerable interest over the past decade.

***Surgery.*** Surgical resection of DF is no longer recommended as a first line treatment option, and it should be reserved for carefully selected patients (9). The high rate of local recurrence, difficulties on achieving negative margins along with the observed high rate of spontaneous tumor regressions are the reasons that led to the progressive decline of upfront surgery (2, 16, 17, 22). Resection can be considered for small DF of the abdominal wall whenever a complete tumor resection is deemed feasible without significant morbidity (21).

***Radiation therapy.*** Radiation therapy is not routinely used in the management of DF and it should be avoided in the young population given the risk of secondary malignancy. Whilst retrospective series have failed to show statistically significant advantages in terms of local control when RT was used in combination with surgery versus surgery alone (23); moderate dose of RT can offer adequate local control (24). Overall, moderate dose RT can be considered in selected cases when systemic treatments are not effective and surgery is not feasible, especially for progressing tumors arising in critical locations as the head and neck region.

***Cryoablation.*** This is a minimally invasive procedure in which a cryoprobe is percutaneously inserted into the tumor to deliver nitrogen or argon gas, inducing the formation of surrounding ice spheres and causing cell death through repeated cycles of freezing and passive thawing (25, 26). This modality of treatment has been increasingly used for DF of the extremity and trunk with several retrospective series showing encouraging data regarding safety and efficacy (25, 27, 28). Recent prospective evidence comes from the phase II clinical trial CRYODESMO-01 which reported that 86% of 50 previously treated patients had non-progressive disease and symptom improvement at 12 months post treatment (29). The vast majority of patients that undergo cryoablation experience grade 1 or 2 toxicity including pain, redness, and swelling confined to the area of treatment, less frequently the formation of an hematoma or transient peripheral nerve damage is observed; serious adverse events are rare and include permanent nerve and neighboring structures or organs damage (29–31).

***High intensity focused ultrasound (HIFU).*** A non-invasive local treatment that uses high frequency ultrasound waves to induce thermal coagulation of the target tissue. The procedure is performed under real time MR thermometry or ultrasound imaging to monitor the energy distribution and ensure sparing of surrounding tissues (32, 33). HIFU ablation is currently approved in the US for the treatment of uterine fibroids (34), prostate cancer (35), and for the treatment of painful bone metastasis (32, 36)with excellent results for symptoms control and functional results (37, 38). Retrospective evidence demonstrated successful employment of this modality of treatment for the management of desmoid fibromatosis (33, 39, 40). Iatrogenic complications of HIFU include grade 1 and 2 skin burns, and temporary nerve injury; less frequent although serious adverse events are ulceration and necrosis of non-target tissue caused by heat conduction and permanent nerve damage (33).

## Medical therapy

Various systemic treatments are available for DF, and with the lack of a defined standard of care, the choice of which agent to use first is left to the treating clinician and institutional experience. **Table 1** illustrates relevant clinical trials evaluating systemic treatment for DF (**Table 1**).

**Table 1:** Main studies reporting on systemic treatment for DF.

Authors/Study [ref]	Year reported	Diagnosis	Phase	Drug	Number of patients	Median age, years (range)	Endpoints	Outcome	p	Key points
Gega M. et al. (41)	2006	FAP associated DF	R	DOX/DTCI iv days 1-4 q28 followed by meloxicam	7	32.2 (28.1-37.1)	Toxicity PFS CR, PR, SD	No G4 74 mo 43%, 57, 0	NA	DOX/DTCI followed by meloxicam is safe and effective
de Camargo V. et al. (42)	2010	DF (32% with Gardner Sd, 44% intra-abdominal)	R	Anthracyclines, hormonal, MTX, imatinib	68	32.5	RR	50% doxorubicin 36% pegylated liposomal doxorubicin	NA	Anthacycline-containing regimens are associated with higher response rate compared to other chemotherapy combinations
Garbay D. et al. (43)	2012	DF (19.5% with Gardner Sd)	R	Mesna, adriamycin, ifosfamide, dacarbazine; Adriamycin, dacarbazine; doxorubicin; etoposide; MTX-vinblastine; vinorelbine; imatinib	62	30 (2–66)	RR CR, PR, SD PFS	54% anthracycline vs 12% 1.6, 19.4, 59.6% PFS 40.8 mo	0.0011	Anthacycline-containing regimens are associated with higher response rate compared to other chemotherapy combinations
Constantinidou A. et al. (44)	2009	DF	R	pegylated liposomal doxorubicin (PLD)	11	29 (3- 53)	PR PFS	36% 14 mo	NA	PLD is effective and has acceptable toxicity profile
Ingley KM. et al. (45)	2019	DF	R	MTX/Vinorelbine	48	33 (13-73)	CR RECIST PR SD PD PFS tolerability	42% 39% 17% 2% 120 mo	NA	Highly effective, sustained response, minimal toxicity
Mir O. et al. (46) Long-term analysis	2020	DF	R	Oral vinorelbine single agent. Oral vinorebine and Hormonal treatment	100	35 (18- 67)	PR SD PFS 6, 12mo	29% 57, 88, 77%	NA	Oral vinorelbine is an effective, affordable, and well-tolerated regimen
Skapek SX. et al. (47)	2007	DF	II	Vinblastine and Methotrexate	26	11.4 (0.6 – 20.4 yo)	PR	31%	NA	Well tolerated. 5/26 pts had G4 neutropenia. First prospective trial in children
Chugh R. et al. (48)	2010	DF	II	Imatinib 300 mg BD orally	51	34 (12- 67)	CBR (clinical benefit rate as CR or PR within 16 weeks or SD lasting 16 weeks at least)	84%	0.24	RR to imatinib is low, PFS prolonged for some patients
Kasper B. et al. (49)	2017	DF	II	Imatinib 800 mg/d orally, treated planned for 2 years	38	44 (19- 80)	PAR_6mo_ PAR_3mo_ ORR	65% 88% 19%	NA	Imatinib induces sustained progression arrest
Gounder MM. et al. (20)	2018	DF	III	Sorafenib 400 mg/d orally	87	37 (28- 50)	PFS	2 y PFS 81% sorafenib vs 36% placebo	< 0.001	Sorafenib significantly prolonged PFS and induced durable responses
Gounder MM. et al. (50) DeFi trial	2022	DF	III	Nirogacestat 150 mg BID, placebo	142	34 (18- 76)	PFS Secondary: safety, ORR, PROs	71% risk reduction vs placebo (HR 0.29)	<0.001	Statistically and clinically significant improvement in PFS, ORR, health related QoL
Gounder MM. et al. (51) RINGSIDE trial Preliminary report	ongoing	DF	II/III	AL102- part A: 1.2 mg QD, 2 mg intermittent BIW (2 days on 5 days off), or 4 mg intermittent BIW	31 in part A, part B currently enrolling	40	PFS	ongoing	NA	ongoing

R, retrospective; DOX, doxorubicin; DTCI, dacarbazine; MTX, methotrexate; TKIs, tyrosine kinase inhibitors; Tem, Temozolomide; Horm, hormonal therapy; PFS, progression free survival; NA, not available; RR, response rate; ORR, overall response rate; CR, complete response; PR, partial response; SD, stable disease; RECIST, Response Evaluation Criteria in Solid Tumors; PAR_6mo_, progression arrest rate at 6 months; PAR_3mo_, progression arrest rate at 3 months; CBR, clinical benefit rate; QoL, quality of life.

***Antihormonal therapy*.** Antihormonal agents s such as tamoxifen or toremifene, alone or in combination with nonsteroidal anti-inflammatory drugs (NSAIDs), have been commonly used to treat DF (52, 53). Their employment was supported by the observed propensity of DF to arise during pregnancy and in the post-partum, and their frequent partial or complete regression after childbirth, supposedly as a consequence of estrogen levels returning to baseline (12, 13, 54–56). The biological rationale for using antihormonal agents comes from the proven estrogen receptor beta expression in 90% of DF (57) and their ability to prevent myofibroblasts differentiation (58). Antihormonal agents showed modest response rate across retrospective series (53, 59). About 30% of patients experience clinical benefit with tamoxifen with no clear correlation with radiological changes on MRI (60). It remains unclear whether the radiological findings and symptomatic improvement are treatment-induced or perhaps expression of the natural course of the disease and whether these drugs could have a role in the treatment of DF, especially when hormone or pregnancy related. Nowadays, antihormonal agents are no longer recommended for the lack of sufficient evidence supporting their use (DTWG).

### Chemotherapy

***Standard chemotherapy.*** Cytotoxic chemotherapy has been long used with evidence of efficacy deriving from several retrospective series and few prospective studies. Anthracycline-based regimes have significant activity in DF with response rate ranging from 37 to 54% (41–43). Patients are generally treated until satisfactory clinical response or when the maximum dose of anthracyclines is reached after 6 to 8 cycles (42). Potential toxicity from treatment include cardiomyopathy, especially when treatment is carried beyond the dose of 450mg/m2, and myelodysplastic syndrome (42). Pegylated liposomal doxorubicin has a reported response rate of 36% and better toxicity profile than its conventional form (44). Overall, anthracycline based chemotherapy regimens are effective and elicit rapid responses but have significant toxicity and should be reserved for selected patients only when a rapid response with prompt symptom control and tumor shrinkage are desired.

***Low dose chemotherapy.*** Low dose chemotherapy with methotrexate (MTX) plus vinblastine (VBL) or vinorelbine (VNL) has been used especially in the young population (46, 47, 61, 62). Disease control is achieved after several months of treatment and response rate ranges between 35 to 40% (63). Late responses occur and contribute to the high long term-disease control with reported median PFS of 75 months and up to 136 months in patients that had responded to treatment (62). Low‐dose MTX/VNL or VBL chemotherapy is effective and minimally toxic regimen but has significant impact on quality of life (QoL) for the lengthy duration of treatment. Single agent oral vinorelbine has a disease control rate of 86% with an excellent toxicity profile (46, 64). Low dose chemotherapy regimens are an effective, safe, and affordable choice that can offer long term symptoms and disease control, however responses are delayed compared to other agents; their use is especially common in the pediatric and young adults’ population for the well understood toxicity profile.

***Tyrosine Kinase Inhibitors.*** The clinical activity of tyrosine kinase inhibitors (TKIs) is well known, and several agents have been investigated in randomized controlled clinical trials. Imatinib, the first TKI evaluated for DF treatment, is effective on achieve disease control with 1 year progression free survival of 66% as confirmed by the results of two separate phase II trial (48, 65). Response to treatment is delayed compared to other agents with best responses seen at 19, 22 and 26 months with decreasing imatinib dosage of 600, 400 or 200 mg per day (48). The overall response rate (ORR) with imatinib is modest and even at the higher dose of 800 mg per day response rate observed is 19% (49). Sorafenib is a multitarget kinase inhibitor whose activity on DF has been extensively studied. The first evidence of efficacy came from the retrospective analysis of a cohort of 24 patients with clinical improvement in 16 (66%) and imaging confirmed partial responses in 5 cases (20%) (66). These observations prompted a more recent phase III placebo-controlled trial of sorafenib 400 mg per day against placebo. The two-year progression free survival was 81% in the treatment arm versus 36% in the placebo arm, while objective response rate for patient on sorafenib was 33% against 20% for placebo, confirming both the activity of sorafenib and quantifying the frequent spontaneous regression observed in DF (20). Pazopanib activity was retrospectively evaluated in a small cohort of 8 patients who received the drug at the starting dose of 800 mg with toxicity-led adjustments and final doses ranging from 200 to 800 mg/day. The overall observed PFS was 13.5 months with PR and SD according to the Response Evaluation Criteria in Solid Tumors (RECIST) 1.1 seen in 3/8 and 5/8 patients respectively (67). DESMOPAZ was a non-comparative, randomized, phase II trial that enrolled patients with DF to receive either pazopanib 800 mg per day or methotrexate and vinblastine chemotherapy. Partial response was seen in 37% of patients with a 6-months PFS of 83%; adverse events led to dose reduction for 73% of patients with fatigue, gastrointestinal toxicity and hypertension being most common (68). In summary, sorafenib and pazopanib are the most effective molecules with sorafenib being often favored in the clinical practice for the milder toxicity profile when compared to pazopanib.

***Gamma secretase inhibitors.*** Recently, new drugs targeting the Wnt/beta-catenin and NOTCH pathways at different levels have been developed with encouraging evidence of efficacy both *in vitro* and *in vivo* (69–72). Reported results from the phase III placebo controlled DeFi trial showed promising activity of the gamma secretase inhibitor (GSI) nirogacestat in patients with progressive desmoid tumors (73). Nirogacestat treatment produced an overall response rate (ORR) of 41%, including 7% complete responses (CR), versus 8% in the placebo arm. Adverse events with nirogacestat were frequent but mostly low grade. Benefit was also measured *via* patient-reported outcomes, including improved pain, stiffness, and functional status (73). This agent is currently undergoing review after New Drug Application submission to the US FDA. Interim results of the phase II/III RINGSIDE trial of the GSI AL102 are also encouraging, showing a favorable toxicity profile and promising preliminary data of effective disease control (51). The beta-catenin inhibitor Tegavivint, which has proven *in vitro* antitumor activity, is currently being investigated in a phase I/II open label trial sponsored by the Children Oncology Group open to patients with recurrent or refractory desmoid tumors as well as other types of solid tumors (NCT04851119) (74, 75).

***Future directions.*** Preclinical studies have implicated the epigenetic regulator EZH2, which is the catalytic subunit of the polycomb repressive complex 2, as a potentially druggable target. They observed *in vivo* inhibition of EHZ2 by tazemetostat with partial regression of autochthonous tumor models and *in vitro* activity of tazemetosat on Wnt pathway (76).

***Areas of uncertainty.*** One of the main open questions remains how to properly select patients for which therapies. Many have postulated that the location of the driver mutation could influence the clinical course of the disease. Three recently reported studies suggested a trend toward worst outcome when the CTNNB1 mutation involves codon 45F, however the correlation failed to reach statistical significance (19, 77, 78). Similarly, mutational status does not correlate with response to treatment, but a correlation with worse general outcome has been observed for APC mutant and non-extremity DF (79).

## Giant cell tumor of bone

Giant cell tumor of bone (GCTB) is a locally aggressive mesenchymal tumor with limited ability to metastasize, low rate of malignant transformation and high local recurrence rate (1).

***Epidemiology.*** GCTB accounts for 3 to 5% of all bone tumors and generally occurs in young adults with peak incidence between 20 and 40 years of age (3, 80).

***Histopathology.*** GCTB has unique histopathological features with a minor subset of stromal mononucleated osteoblast-like cells that are thought to be responsible for the growth and survival of a second population of multinucleated, osteoclast-like giant cells. The neoplastic stromal osteoblastic cells produce chemotactic factors including nuclear factor kappa B ligand (RANKL). Increased levels of RANKL promote pathologic recruitment of monocytes to the tumor site and induce their differentiation into osteoclasts-like giant cells, ultimately responsible for the osteolysis seen in GCTB (**Figures 1C, D**) (81–83).

***Tumor classification.*** There are two subtypes of GCTB: a more common conventional type, and a primary malignant GCTB, a rare entity accounting to less than 2% of new diagnoses (84, 85). Moreover, 2 to 3% of conventional GCTB can undergo sarcomatous transformation into a malignant tumor, in most cases several years after radiotherapy or curettage (86–88). Conventional GCTB can rarely metastasize, this occurs in less than 10% of patients with the lung being the most common site of secondary disease. Pulmonary involvement tends to remains asymptomatic, and it is not necessarily linked to malignant transformation (89).

***Clinical presentation.*** GCTB predominantly arises from long bones such as femur and tibia, especially around the knee, but it can also affect the pelvis, smaller bones of feet and hands, and other less typical locations (90). Clinically, GCTB can cause pain, swelling, deformity, and loss of function depending on the site of disease; if left untreated, GTCB can lead to bone resorption, fracture, and neurological symptoms (91, 92).

***Local treatments.*** The mainstay of treatment for GCTB is aggressive curettage or surgical en-bloc resection of the affected bone, while medical treatment is reserved for recurring or unresectable tumors and in lieu of morbid surgical procedures (92). Intralesional curettage with allograft or bone cement reconstruction is a widely accepted procedure that allows local control without sacrificing function (91–93). Local recurrence rate after curettage is high, ranging between 25 and 50% with conflicting reported data regarding the impact of different bone reconstruction techniques and filling materials (91, 92, 94–101). Peri-surgical interventions have been explored with the intent to lower the rate of local recurrence with no evidence of benefit so far (102, 103). Adjuvant radiotherapy may decrease the chances of post-surgical recurrence, but it is known to induce secondary malignant transformation, making it not a commonly pursued treatment (86, 87, 104–106).

## Medical treatment

***RANK ligand inhibitors.*** Denosumab is a fully human IgG2 monoclonal antibody that binds RANKL, preventing it to interact with his receptor, RANK, on the surface of osteoclasts and their precursors. Reduced RANL-RANK binding inhibits osteoclasts formation, function, and survival, ultimately controlling osteolysis and inducing ossification and fibrosis (107–111). The first proof of concept study of denosumab on GCTB was an open-label phase II study that enrolled 37 patients with recurrent or unresectable tumors to receive subcutaneous denosumab 120 mg every 4 weeks with additional doses on day 8 and 15. The primary endpoint was the proportion of patients with a tumor response at 25 weeks defined as histopathological confirmed elimination of 90% of giant cells; or, where giant cells represented less than 5% of tumor cells at baseline, complete elimination. Non radiological progression was used to estimate efficacy when histopathologic data were not available. Of 35 assessable patients, 30 had either histological or radiological response (112). Later analysis of tumor specimens confirmed that denosumab significantly reduces RANK-positive tumor giant cells, as well as the relative proportion of proliferative, densely cellular tumor stroma, and promotes the formation of differentiated bone tissue (108). A larger phase II study enrolled 282 patients distributed in three cohorts to receive denosumab at the established dose of 120mg subcutaneously every 4 weeks with extra doses on day 8 and 15 of the first cycle. Patients in cohort 1 (n = 169) had inoperable disease and received denosumab as the only treatment. Patients in cohort 2 (n= 100) received neoadjuvant denosumab for salvageable GCTB, these patients had GCTB that were deemed resectable with technically feasible, but potentially morbid surgical resections. Cohort 3 included patients who were transitioned from the previous phase II study. Results from interim analysis after a median follow up of 13 months showed that 96% of patients from cohort 1 had non-progressive disease; seventy-four percent of patients from cohort 2 had not undergone surgery and, among the 26 patients who did, 16 had a less morbid procedure than initially planned. Toxicity included joint pain in 20% of patients followed by headache, nausea, back pain, and fatigue; osteonecrosis of the jaw (ONJ) was seen in 1% of patients (113). Based on demonstrated efficacy, denosumab was approved by the FDA in June 2013 for its use in adults and skeletally mature adolescents with giant cell tumor of bone deemed unresectable or requiring morbid surgery or in metastatic disease. Long term follow up data of the same trial was analyzed for safety and efficacy and published in 2019, after the enrollment was expanded to include a total of 532 patients. The median follow up was 58 months for the overall population, 65.8 months for cohort 1 and 53.4 months for cohort 2; at the time of the analysis 11% of patients in cohort 1 had progressed and 92% of patients in cohort 2 had not undergone surgery in the first 6 months of treatment. Common G3 or G4 toxicity were hypophosphatemia (5%), osteonecrosis of the jaw (ONJ) (3%), atypical femoral fracture (1%); 1% of patients presented with hypercalcemia occurring 30 days after discontinuing treatment (114).

The role of denosumab in the neoadjuvant setting has been also evaluated, with conflicting results so far (102, 103, 115). A later analysis of the expanded cohort 2 of the above-mentioned trial evaluated the potential impact of pre-operative denosumab on downstaging surgery. A total of 222 patients candidate to extensive surgeries (hemipelvectomy, amputation, joint replacement/fixation) were treated with denosumab for a median duration of 15.3 months; at the date of cutoff for data analysis, 48% of patients had not yet undergone surgery, while 38% of them had been able to undergo less morbid surgeries than originally planned. In this study, 17 of the 116 surgical patients experienced disease recurrence after a median time of 13.6 months. Notably, the median follow up post-surgery was 13 months, hence the results may underestimate the actual rate of local recurrence (116). Further evidence supports the use of denosumab for patients with unresectable disease as well as in the neoadjuvant setting, as it may facilitate surgery and allow avoidance of mutilating resections (117–119).

Data suggests that the combination of neoadjuvant denosumab and curettage is associated with a high risk of disease recurrence. Errani et al. reported local recurrence rate as high as 60% (15/25) for patients who underwent curettage after receiving denosumab versus 16% recurrence rate (36/222) for patients treated with upfront curettage (120). This was confirmed by several groups and by a recent metanalysis that showed that tumors treated with denosumab plus curettage have a relatively higher risk of recurrence compared with tumors managed with curettage alone (P = 0.07) (102). It has been postulated that denosumab-induced tumor changes may be responsible of the higher recurrence rate; for example, the development of a peripheral calcified rim that can preclude radical curettage as well as the persistence of latent tumor cells in the new formed bone may represent the cause of recurrence and may require more aggressive curettage (108, 121, 122). This is supported by the knowledge that denosumab targets the osteoclastic cells and lacks antitumor effect against neoplastic stromal cells that can restart proliferating when the RANKL Ab disappears from the microenvironment, as proven by *in vitro* evidence (123).

The short-term efficacy of denosumab and toxicity profile at the standard dosing in patients with unresectable GCTB are well described. However, patients with unresectable GCTB are, by definition, candidates for prolonged treatment that can lead to drug related complications. On the other hand, discontinuation may be followed by disease relapse (124). In a cohort of 54 patients with unoperable or metastatic GCTB, ONJ was observed in 9% of patients, skin rash and hypophosphatemia in 11 and 4% respectively. Ten patients discontinued denosumab and were followed up for a median time of 15 months; 4 of them had disease progression after 7 to 15 months from treatment discontinuation, while 6 had no signs of active disease months to a few years after treatment cessation (124). Rebound hypercalcemia with acute kidney injury 5.5 to 7 months post denosumab was described in three young patients (14, 15 and 40 years) who had been treated for 1.3 to 4 years and stopped treatment for toxicity (125). This prompts new questions regarding the optimal length of treatment with evidence that some patients may be able to discontinue denosumab and enjoy sustained response, while other may need longer treatment (124, 126).

Increasing interval of denosumab dosing and establishing the optimal length of treatment may help find a balance between satisfactory disease control and avoidance of serious adverse events. Effects of increased dosing interval has been evaluated in a retrospective cohort of 37 patients. Dosing interval was increased for 38% of patients with most common final interval of 12 weeks, this resulted in similar tumor control compared to standard dosing and lower absolute number of bone toxicity events (127). The rationale supporting longer interval is that the half-life of denosumab is 32 days and the inhibitory effects on osteolysis lasts 12 weeks (128, 129). The REDUCE trial, whose results are awaited, was designed to investigate risks and benefits of maintenance treatment with reduced intensity denosumab after 12-15 months of conventional dose treatment in patients needing long term therapy (130). Overall, denosumab provides long term disease control for patients with unoperable GCTB and its use is now well established. Conversely, the decision of initiating medical treatment for patients with operable GCTB should be pondered and the selected surgical modality defined prior to the start of systemic treatment. In fact, although denosumab may improve the outcome for patients undergoing en-bloc resections, it can increase the risk of local recurrence in case of intralesional curettage. Therefore, surgical and medical treatment planning for GCTB should be coordinated by a sarcoma multidisciplinary team.

***Tyrosine Kinase Inhibitors.*** Lenvatinib is a multitargeted tyrosine kinase inhibitor whose effect on GCTB patient derived 2D and 3D primary culture was tested in a recently reported study. Five patients derived primary GCTB series were exposed to denosumab, lenvatinib and a combination of denosumab and lenvatinib. Interestingly, lenvatinib exhibited higher activity both in 2D and 3D compared to denosumab (131). The involvement of VEGFR has been described in supporting RANKL-induced osteoclastogenesis in GCTB and the above results confirm the promising role of antiangiogenic drugs in its management (131, 132). **Table 2** illustrates relevant clinic trial assessing systemic treatment for GCT (**Table 2**).

**Table 2:** Main studies reporting on systemic treatment for GCTB.

Authors/Study [ref]	Year reported	Diagnosis	Phase	Drug	Number of patients	Median age, years (range)	Endpoints	Outcome	p	Key points
Thomas et al. (112) First study of denosumab for GCT	2010	GCTB	II	Denosumab 120 mg sc q4w with extra dose day 8 and 15	37	34 (22- 46)	RR at 25 weeks (elimination of at least 90% of giant cells or no radiological progression of the target lesion)	86% 30/35 responders 20/20 histologically 10/15 radiologically	NA	Denosumab elicits histological and radiological response
Chawla et al. (113) Interim analysis	2013	GCTB	II	Denosumab 120 mg sc q4w with extra dose day 8 and 15	282 in 3 cohorts	33.5	safety profile TTP -cohort1 Time to surgery -cohort2	96% at 13 months 74% no surgery at 9.2 months	NA	Denosumab was associated with tumour responses and reduced the need for morbid surgery
Martin-Broto et al. (6) Interim results of the previous study	2014	GCTB	II	Denosumab 120 mg sc q4w with extra dose day 8 and 15	281	33.5	Proportion of patients with clinically relevant decrease in worst pain Time to decrease in pain Time to increase in pain	Cohort 1,2, and 3: 29% and 35% 77% and 79% 30 and 15 days 6.9 and 30%23.2 months- N/A	NA	Rapid and clinically relevant pain relief
Rutkowski et al. (116) Analysis of cohort 2 from phase 2 trial from Chawla 2013	2015	GCTB	II	Denosumab 120 mg sc q4w with extra dose day 8 and 15	222	34 (25-44)	Cohort 2 patients for surgical downstaging rate	48% had no yet undergone surgery at cutoff time; 38% had less morbid surgeries	NA	Beneficial surgical downstaging, including either no surgery or a less morbid surgical procedure
Chawla S. et al. (114) Long term follow up	2019	GCTB	II	Denosumab 120 mg sc q4w with extra dose day 8 and 15	532	33 (25- 45)	Primary: Safety Secondary: PFS for cohort 1; percentage of patients not undergoing surgery for cohort 2	PFS not reached at the preliminary analysis	NA	Denosumab is safe and shows long term disease control
Bukata et al. (133) Subanalysis of phase II from Chawla 2013	2019	GCTB of the spine including sacrum	II	Denosumab 120 mg sc q4w with extra dose day 8 and 15	132	32 (13–83)	Safety Efficacy with estimate of PFS For patients in cohort 1	- 3% and 7.4% at 1 and 3, 5 years	NA	Safe and potentially useful for GCTB of spine and sacrum
ClinicalTrials.gov Identifier: NCT03620149	2021	GCTB	II	Denosumab maintenance 120mg SC 12-weekly (after 12-15 months at conventional dosing)	NA	NA	PFS ONJ	Not reported	Not reported	Not reported
Jiang et al. (127)	2022	GCTB	R	Denosumab 120 mg sc at various increased interval, most commonly 12 weeks	37	37 (22-73)	Difference in efficacy and bone toxicity or standard dose vs increased interval Median PFS 5-year PFS	No difference in efficacy, toxicity, mPFS. 5 y PFS was longer for less frequent dosing	NA p= 0.22 p= 0.97 p= 0.036	Tumor control is similar, bone toxicity is better with enlarged intervals

R, retrospective; PFS, progression free survival; mPFS, median progression free survival; RR, response rate; ORR, overall response rate; CR, complete response; PR, partial response; SD, stable disease; RECIST, Response Evaluation Criteria in Solid Tumors; CBR, clinical benefit rate; QoL, quality of life; NA, not available.

## Malignant giant cell tumor of bone

GCTB can rarely undergo malignant transformation and acquire histopathological characteristics that are similar to a high-grade sarcoma such as undifferentiated sarcoma or osteosarcoma (145). Malignant transformation is reported in 1 to 4% of patients; malignant GCTB are classified as primary malignant (PMGCTB), secondary malignant GCTB (SMGCTB) or GCTB with sarcomatous transformation not secondary to treatment (84, 146). In primary malignant GCTB, distinct areas of benign GCTB are juxtaposed with high-grade sarcoma ones, making it a challenging and often missed diagnosis (85, 147). The radiologic features of PMGCTB are also similar to those of conventional GCTB presenting as osteolytic lesions with well-circumscribed margins (84, 85, 148). In secondary malignant GCTB (SMGCTB), malignancy is diagnosed at the site of conventional GCTB previously treated with radiation or surgery (147, 148). Malignant transformation in GCTB after or during treatment with denosumab has also been reported; however it remains unclear whether denosumab can favor malignant transformation through immunosuppression or if at least some progressive SMGCTB were malignant tumors initially misdiagnosed (111, 113, 114, 116, 146, 149–152). Sarcomatous transformation of conventional, treatment naïve GCTB has been sporadically observed (146). Latency between the primary diagnosis of conventional GCTB and malignant GCTB can vary between 3 to over 20 years according to historical data (146, 147, 153).

Overall, malignant GCTB is associated with poor outcome, with post-radiation SMGCTB showing an especially aggressive behavior (147, 153). Malignancy should be suspected in case of pulmonary involvement, poor response to denosumab, aggressive clinical behavior and disease that recurs after a latency period of more than 4 years (84, 148). Surgical resection is the mainstay of treatment for malignant GCBT (154). Although adjuvant chemotherapy has failed to improve the overall survival for patients with malignant GCBT, it seems associated with longer pulmonary metastasis free survival (148).

## Metastatic GCTB

Metastatic disease is rare and typically involves the lungs. The pathophysiology of pulmonary metastasis of GCT has not been determined, and various factors from tumor vascular invasion to iatrogenic embolization have been suggested as the cause pulmonary spread (155). Pulmonary metastases have matching histological features to the primary tumor, are generally indolent and not necessarily linked to malignant transformation, however the incidence of lung metastasis is high for malignant GCTB (148, 156). The observed interval between primary diagnosis and development of pulmonary metastasis is significantly shorter for malignant GCTB compared to the conventional type (9 vs 21 months) according to a large retrospective case series reported by Liu et al. (148). The incidence of lung metastasis seem to be influenced by the presence of malignancy, time to recurrence, time for primary diagnosis and tumor size (157). The clinical course of pulmonary metastatic disease is unpredictable. Lung metastasis may be managed with surveillance at first, however about 50% of patients will eventually experience disease progression and need treatment with metastasectomy or denosumab (158). Overall, the prognosis of patients with metastatic disease is favorable but many questions remain open including surveillance recommendations, risk stratification and best management of disease.

## Tenosynovial Giant Cell Tumor

Tenosynovial Giant Cell Tumor (TSGCT) is a rare, locally aggressive neoplasm that arises from the synovium of joints, bursae, and tendon sheaths (4, 6).

***Epidemiology.*** The incidence of TSGCT is estimated to be of 1.8 cases per million per year in the USA, with a peak between 30 and 50 years of age and female prevalence (3, 159–161).

***Histopathology.*** TSGCT is characterized by elevated expression of the colony-stimulating factor (CSF1) gene (97). Several mechanisms leading to CSF1 hyperexpression have been described such as translocations or deletions, the vast majority of them resulting on exon 9 deletion, which negatively regulates CSF1 expression (134, 162, 163). This causes overexpression of CSF1, responsible for the recruitment and growth of CSF1R expressing monocytes and drives the development of a tumor formed by a large number of nonneoplastic macrophages expressing CSF1R and a minority of neoplastic cells, which do not express CSF1R (134, 162–164) (**Figures 1E, F**).

***Tumor classification.*** TSGCTs are classified in two distinct subtypes based on growth pattern and presentation: localized or nodular type (N-TSGCT) and infiltrative diffuse type (D-TSGCT). Although D-TSGCT displays an infiltrative border, both subtypes are strikingly similar microscopically, being comprised of an admixture of cell types without significant cytologic atypia (**Figures 1E, F**). N-TCGT, the most common subtype, arises from digits in 80% of cases with less frequent locations being the wrist, ankle, foot, knee and, even more rarely, large joints. D-TCGT is rare and affects the knee in 75% of observed cases, followed by the hip, elbow, shoulder and ankle (81). An extra-articular form D-TSGCT is possible, with tumor growth within the peri-articular soft tissue and no evidence of articular involvement (165). Malignant TSGCT is exceedingly rare and affects people between 50 and 60 years of age; is characterized by areas of sarcomatous differentiation and tends to metastasize to the lymph nodes and lungs rather than locally recur (161, 166–170).

***Clinic and natural history.*** TSGCT has an excellent prognosis, and, with the exception of the rare malignant form, it is not considered a life-threatening disease (171). Clinically, N-TSGCT tends to have an indolent course, while D-TSGCT is more aggressive and can have variable behavior from paucisymptomatic to severely symptomatic disease with joint pain, swelling, locking, instability, numbness, diminished range of motion and decreased quality of life. Not all patients experience symptoms, and for this reason management should be individualized and the clinical presentation must be considered when deciding between active surveillance versus systemic or surgical treatment (172, 173).

## Local treatments

***Surgery.*** In case of symptomatic disease, surgery is the primary treatment for both subtypes. However, there is growing consensus on wanting to avoid morbid resections and consider systemic treatment instead (6). Most N-TSGCT can be cured with marginal resection, whilst D-TSGCT require extensive synovectomy and, despite this, have a chance of local recurrence reported between 30 and 50% with even higher rate for repeat resections (171).

***Radiation therapy.*** Peri-operative interventions with systemic treatment or radiotherapy are not standard of care although considered by some authors for borderline operable cases (174).

## Medical treatment

***CSF1R inhibitors.*** Improved insight into tumor biology has revolutionized systemic treatment and several molecules targeting CSF1/CSF1R have successfully been employed. Pexidartinib is an orally available CSF1R inhibitor approved in the USA for the treatment of adults with inoperable and severely debilitating tumors (164, 175). Evidence that brought to the approval of pexidartinib comes from a phase III study against placebo showing an overall response rate of 39% in the treatment arm at week 25 versus 0% for placebo, as well as improvement in patient-reported outcomes, including scores for pain, stiffness, and function (135, 175). To assess the long-term effects of pexidartinib, a pooled analysis of studies ENLIVEN and the TSGCT cohort of the PLX 108-01 study was performed by Gelderbom et al. (136). The study population consisted of a cohort of 120 patients treated with pexidartinib; ORR was 60% according to RECIST and 65% according to Tumor Volume Score (TVS) measurement, 77% of responses occurred within 6 months from treatment start, and the median duration of treatment was 19 months. Regarding toxicity, 68% of patients experienced adverse events (AEs) requiring dose reductions or treatment discontinuation; 92% had aminotransferase elevation between 1 and 3 x ULN in 66% of cases, while 4 patients had mixed cholestatic hepatotoxicity which resolved within 1 to 7 months from drug interruption (136).

***Imatinib.*** Imatinib mesylate blocks the CSF1R and is active against TSGCT. Evidence of efficacy comes from a large multicenter retrospective study that included 58 patients treated with imatinib for advanced symptomatic, recurrent, or metastatic (2 patients) TSGCT. The response rate (RR) among all patient was 31%, PFS was 18 months, patient reported clinical benefit was favorable as well as the toxicity profile (137, 143).

***Nilotinib.*** Nilotinib, a tyrosine kinase inhibitor active against CSF1, has shown short-term disease control with 90% of PFS at 12 weeks, and mixed long-term disease control with PFS of 52% at 5 years (139, 140). Further data from a recently completed phase II study of Nilotinib in patients with relapsed or metastatic TSGCT are awaited and will help clarify the role of Nilotinib for TSGCT treatment (NCT01207492).

***Ongoing clinical trials.*** New agents are also currently being studied in ongoing clinical trials. ***CSF1R inhibitors.*** ViImseltinib is an oral CSF1 inhibitor currently investigated on the ongoing phase III MOTION trial (NCT05059262). Recently reported results from phase I and phase II trials show that all enrolled patients benefited from treatment in terms of symptoms or disease control with manageable toxicity profile (144, 176).

***Monoclonal antibodies against CSF1R.*** Monoclonal antibodies against CSF1R cabiralizumab and emactuzumab have been studied on patients with D-TSGCT with preliminary evidence of efficacy from phase I/II trials (138, 141, 142). Results from a recently completed phase III trial of emactuzumab are awaited (NCT05417789).

Class effect toxicities of CSF1/CSF1R inhibitor including hypertension, oedema, and liver toxicity can rarely be serious. In the attempt to avoid systemic toxicity and successfully treat this localized disease, a trial of intra-articular administration of the CSF1 receptor antibody AMB-05X is ongoing (NCT05349643). (**Table 3**) illustrates relevant clinic trial assessing systemic treatment for TSGCT.

**Table 3:** Main studies reporting on systemic treatment for TSGCT.

Authors/Study [ref]	Year reported	Diagnosis	Phase	Drug	Number of patients	Median age, years (range)	Endpoints	Outcome	p	Key points
Tap W. et al. (134)	2015	TSGCT	I/II	Pexidartinib po 1000mg/d	20	46 (22- 80)	Clinical benefit CR/PR/SD	95% 0/12/7	NA	Prolonged regression of tumor
Tap W. et al. (135) ENLIVEN	2019	TSGCT	III	Pexidartinib po 1000mg/d vs placebo po	120	44 (22- 75)	RR at 25 weeks CR/PR	39% vs 0 15%/25%	0.0001	Robust tumor response with improved symptoms; mixed or cholestatic hepatotoxicity is an identified risk.
Gelderblom et al. (136) Long term effects of pexidartinib	2021	TSGCT	Pexidartinib pooled analysis	Pexidartinib po 800-1000mg/d	130	45 (20- 80)	Best overall response by RECIST (CR, PR) DOR by RECIST	78% Not reached	NA	Overall LT benefit of continued treatment with pexidartinib
Cassier PA. et al. (137)	2012	TSGCT	R	Imatinib 400 mg/day orally	27	41 (21- 77)	RR SD CR/PR	19% 74% 1/4	NA	Potential effect of imatinib on targeting CSF1
Cassier PA. et al. (138) Emactuzumab Phase I long-term	2015	TSGCT	I	Emactuzumab IV 900-2000mg/2weeks	28	42 (18- 82)	Safety RR CR	86% 7%	NA	Promising activity, 5 serious adverse events
Gelderblom H et al. (139)	2018	TSGCT	II	Nilotinib 800mg/day orally	56	36 (18- 74)	PFS 12 weeks	94%	NA	Manageable toxicity and good disease control at 12 weeks
Spierenburg G et al. (140) Long-term Nilotinib Ph II	2022	TSGCT	II	Nilotinib 800mg/day orally	48	37 (23- 51)	LT-PFS Duration of response mTTP clinical worsening LT- toxicity	48%	NA	Mixed effect of nilotinib with half of patient needing nire treatment at 8.5 years follow up
Sankhara KK. et al. (141)	2017	D-TSGCT	I/II	Cabiralizumab 1, 2, 4mg/kg	22	not reported	Safety efficacy	not reported	Not reported	Not reported
Cassier PA. et al. (142)	2020	D-TSGCT	I	Emactuzumab IV 900-2000mg/2weeks	63	38 (18–82)	Safety RR CR/PR	71% 3%, 68%	NA	Manageable toxicity, durable response
Verspoor FGM et al. (143)	2019	TSGCT	R	Imatinib 400 mg/day orally	58	45 (36–56)	RR CR/SD PFS 1 and 5 years	31% 4%, 27% 71 and 48%	NA	Prolonged responses even after treatment discontinuation
Blay JY et al. (144)	2022	TSGCT	II	Vimseltinib 30 mg twice weekly	57 (46 cohort A, 11 cohort B)	45 (21–71)	Safety RR CB (PR+SD)	49% cohort A 44% cohort B CB 100%	NA	Manageable toxicity, effective
MOTION trial NCT05059262	ongoing	TSGCT	III	Vimseltinib 30 mg twice a week	Ongoing	18 yo or older	ORR at 25 weeks ORR per tumor volume score PROs	Ongoing	–	Ongoing

R, retrospective; PFS, progression free survival; RR, response rate; ORR, overall response rate; CR, complete response; PR, partial response; SD, stable disease; CBR, clinical benefit rate; RECIST, Response Evaluation Criteria in Solid Tumors; DOR, duration of response; LT, long term; NA, not available.

## Discussion

Despite progress made in systemic and local treatments and improved understanding of disease biology, patients with locally aggressive mesenchymal tumors still may experience unsatisfactory outcomes and detriment to quality of life. Treatment paradigms still vary, given the rarity of these diseases and lack of consensus guidelines. Misdiagnoses are frequent and contribute to suboptimal management, worse outcome, and inadequate patient experience (2, 177, 178). All this is being improved thanks to *ad-hoc* instituted working groups and joint effort of scientists and patient associations across the world. For example, a global consensus meeting held in 2018 brought together world experts and started the process of defining a standard of care for DF. Practice changing conclusions were reached such as the recommendation to proceed with a period of active surveillance for newly diagnosed DF and to consider medical treatment as first option rather than surgery (9). Prospectively controlled clinical trials require partnership and are critical to validating future treatment recommendations for these and other locally aggressive mesenchymal neoplasms. Many efforts have been made in the past few years to identify prognostic and predictive biomarkers and select the best candidates and potential responders to treatment. Recently published studies have significantly contributed to the understanding of the natural history and potential prognostic significance of mutational status in DF. Although no association reached statistical significance, a trend toward worst outcome for tumors harboring mutations involving codon 45F of the CTNNB1 gene, for APC mutated DF and for non-extremity site of disease was uncovered (19, 77, 79). There are not known prognostic factors for GCT or TSGC that can help stratify patients. Massive parallel sequencing of 34 resection specimens of TSGCT detected the presence of a CBL missense mutation in 35% of tumors which was significantly associated with shorter time to local recurrence (179).

Complexity is added to the management of locally aggressive mesenchymal tumors by the unsatisfactory correlation between RECIST assessment and treatment effectiveness. As postulated by many, a better surrogate of treatment efficacy may be the change of T2 signal on MRI; a shift from long to short T2 signal has been in fact observed in DF when tumor histology transitioned from more cellular to more fibrous, hypocellular tissue (180). Similarly, for GCT, RECIST assessment is not an accurate measure of treatment efficacy and the use of modified PET scan criteria or inverse Choi density/size criteria have been proposed to assess response to denosumab (181). Comparable limitations challenge response assessment for TSGCT for which a volumetric comparison of the tumor pre and post treatment may represent a more precise way of measurement than diameter comparison, given the irregular shape of the tumor (6).

Finally, how to select the patients that may benefit the most from treatment and for how long to treat are crucial points that need to be addressed. The newly introduced drugs have shed some light, but they have also uncovered the very specific challenge of exposing patients with non-malignant conditions to the risk of potential long-term toxicity. Many aspects that go beyond the disease itself warrant careful consideration. These patients report persistent pain, emotional distress, and financial hardship (182, 183). While these are non-malignant diseases, the long-term effects of treatments and impact on quality of life resemble cancer in many ways. Patient reported outcomes are a necessary tool to finally strike a balance between the desirable disease control and other non-negotiable aspects such as family planning, ability to work, financial wellness, and good overall quality of life (8, 172, 173). Given the rarity of this class of tumors, complex patient needs, and to avoid suboptimal outcomes, treatment planning should be individualized and planned in the context of an expert multidisciplinary team.

## Author contributions

AM drafting, writing, editing the manuscript, collecting and analyzing data. JZ collecting and analysing data. MC data collection, editing the manuscript. BW collecting and analysing data, editing the manuscript. All authors contributed to the article and approved the submitted version.
